# Amantadine sulfate treatment in cases with brain injury in the ICU: a retrospective clinical trial

**DOI:** 10.1186/cc14533

**Published:** 2015-03-16

**Authors:** S Goksu Tomruk, N Bakan, G Karaören, A Salvarcý

**Affiliations:** 1 Umraniye Research and Training Hospital, Istanbul, Turkey

## Introduction

Improvement of recovery is a challenging process in cases with varying degrees of severe brain injury (BI) requiring intensive care. Amantadine sulfate (AS) is recommended for use in cases with brain injury. The Coma Remission Scale (JFK-CRS) consists of auditory-visual-motor-mouth-tongue functions, communication and awareness scales; provides a score between 0 and 23; and shows numeric recovery from coma. The aim of this study was to evaluate outcomes and effects of AS used for neurorecovery on the Glasgow Coma Scale (GCS) and JFK-CRS in our ICUs during the last 5 months.

## Methods

After approval of the Ethics Committee, we recruited 12 patients with brain injury resulted from trauma or hemorrhage who had initial GCS of ≤9 and received AS (500 mg, twice daily over 10 days) during the recovery period. In all subjects, age, gender, diagnosis, initial APACHE II score, time of initiation of AS therapy, JFK-CRS and GCS scores, aspartate aminotransferase, alanine aminotransferase, BUN, creatinine, platelet count, electrocardiography findings, electrolyte values and arterial blood gas values on days 1, 6, 10 and 14 were recorded.

## Results

The patients' diagnoses included two post-CPR, five intracranial and one subdural hematoma, one CVA, one postoperative aneurysm, one subarachnoid hemorrhage and one brain contusion. Table 1 presents the findings. The AS therapy was initiated between days 3 and 33 of admission in all patients other than Patients 2 and 8. A dramatic improvement was observed in a patient with both GCS and JFK-CRS score of 5 when AS therapy was initiated in month 5 and patient was discharged for home care. In Patient 9, AS therapy was withdrawn on day 5 due to persistent thrombocytopenia (TP) despite exclusion of other reasons; subsequently, improvement was observed in TP. The complications were relatively less severe with average acceptability.

**Table 1:** Findings of 12 patients receiving AS.

Initiation of Patient number AS therapy (day)	Sex/age (years) CRS score D/E/F/G	APACHE II Complications	GCS A/B/C Outcome
1	M/14	24	3/5/7
6	0/4/5/6	H, J	Still in ICU
2	F/78	33	8/8/10
84	0/0/0/0	K	Ex
3	F/75	35	5/6/4
4	1/1/0/Ex	K, L	Ex
4	M/24	29	3/3/3
6	0/0/Ex	K, L	Ex
5	M/27	27	4/4/15
6	1/13/18/22	L, M, J	Discharged
6	F/70	45	3/3/3
20	0/0/0/0	-	Ex
7	F/72	22	5/5/12
3	4/7/11/17	K, L, I	Discharged
8	F/53	27	3/5/10
150	5/7/10/14	K	Discharged
9	F/38	34	4/4/4
33	0/0/1/1	H, J	Still in ICU
10	M/50	26	4/6/13
8	0/7/18/19	L, J	Discharged
11	M/63	24	8/10/12
14	6/8/8/12	-	Discharged
12	M/50	31	4/6/11
6	2/5/7/9	L, J	Still in ICU

F, female; M, male. GCS, Glasgow Coma Scale: A, at admission to ICU; B, at initiation of AS therapy; C, outcome value. CRS: D, value on day 1, E, value on day 6, F, value on day 10, G, value on day 14. Complications observed: H, low platelet; J, ALT increase by twofold; K, BUN increase by twofold; L, AST increase by twofold; M, convulsion.

## Conclusion

We suggest that an AS dose of 1,000 mg/day (over 10 days) seems to improve neurorecovery in BI patients with good tolerability. Prospective controlled studies with large, homogeneous BI populations will better define the role of AS for recovery and complications.

